# Using extensional flow to reveal diverse aggregation landscapes for three IgG1 molecules

**DOI:** 10.1002/bit.26543

**Published:** 2018-02-04

**Authors:** Leon F. Willis, Amit Kumar, John Dobson, Nicholas J. Bond, David Lowe, Richard Turner, Sheena E. Radford, Nikil Kapur, David J. Brockwell

**Affiliations:** ^1^ Astbury Centre for Structural Molecular Biology University of Leeds Leeds West Yorkshire UK; ^2^ School of Molecular and Cellular Biology, Faculty of Biological Sciences University of Leeds Leeds West Yorkshire UK; ^3^ School of Mechanical Engineering Faculty of Engineering University of Leeds Leeds West Yorkshire UK; ^4^ MedImmune Ltd Granta Park Cambridge UK

**Keywords:** aggregation, antibody, bioprocessing, extensional flow, shear flow

## Abstract

Monoclonal antibodies (mAbs) currently dominate the biopharmaceutical sector due to their potency and efficacy against a range of disease targets. These proteinaceous therapeutics are, however, susceptible to unfolding, mis‐folding, and aggregation by environmental perturbations. Aggregation thus poses an enormous challenge to biopharmaceutical development, production, formulation, and storage. Hydrodynamic forces have also been linked to aggregation, but the ability of different flow fields (e.g., shear and extensional flow) to trigger aggregation has remained unclear. To address this question, we previously developed a device that allows the degree of extensional flow to be controlled. Using this device we demonstrated that mAbs are particularly sensitive to the force exerted as a result of this flow‐field. Here, to investigate the utility of this device to bio‐process/biopharmaceutical development, we quantify the effects of the flow field and protein concentration on the aggregation of three mAbs. We show that the response surface of mAbs is distinct from that of bovine serum albumin (BSA) and also that mAbs of similar sequence display diverse sensitivity to hydrodynamic flow. Finally, we show that flow‐induced aggregation of each mAb is ameliorated by different buffers, opening up the possibility of using the device as a formulation tool. Perturbation of the native state by extensional flow may thus allow identification of aggregation‐resistant mAb candidates, their bio‐process parameters and formulation to be optimized earlier in the drug‐discovery pipeline using sub‐milligram quantities of material.

## 1 INTRODUCTION

The advent of hybridoma (Kohler & Milstein, 1975) and phage display technologies (Winter, Griffiths, Hawkins, & Hoogenboom, 1994) has allowed monoclonal antibodies (mAbs) to revolutionize the biotechnology industry (Aggarwal, 2014). Since the approval of Orthoclone OTK3 in 1986, over 50 mAb‐based biologics have been launched, comprising half of the $140 billion biopharmaceutical market (Chiu & Gilliland, 2016; Elgundi, Reslan, Cruz, Sifniotis, & Kayser, 2017). The ability to generate high affinity candidates to a broad range of targets, together with the development of multi‐partite therapeutic strategies, has rendered mAbs and their derivatives important scaffolds (Aggarwal, 2014).

One barrier that slows or even halts the process of bringing mAbs to market is aggregation (Buss, Henderson, McFarlane, Shenton, & de Haan, 2012) due to unfolding (partial or complete) or mis‐folding (Mahler, Friess, Grauschopf, & Kiese, 2009; Roberts, 2014). While generally deleterious to protein function, aggregation is particularly problematic to the biopharmaceutical industry as aggregates have been linked to immunogenic reactions in patients (Büttel et al., 2011) and to shortened therapeutic half‐life (Dobson et al., 2016). In addition, the presence of aggregates during development can lead to a decreased yield and an increase in time to market, due to the need to optimize manufacturing conditions/formulation (Cromwell, Hilario, & Jacobson, 2006; Zurdo et al., 2015). mAb‐based biologics are susceptible to aggregation throughout their lifetime, from over‐expression in the cell (Kramarczyk, Kelley, & Coffman, 2008) and downstream processing (Skamris et al., 2016; Yu et al., 2016) to the final fill‐finish step at high concentration (Cromwell et al., 2006; Rathore & Rajan, 2008). In contrast to the effects of temperature and pH (reviewed in Roberts, 2014), the effects of biopharmaceutical manufacture and transport stresses, such as air‐water interfaces (Bee et al., 2012; Maa & Hsu, 1997) and hydrodynamic forces (Biddlecombe et al., 2007; Brückl, Schröder, Scheler, Hahn, & Sonderegger, 2016), are relatively under‐studied (Bekard, Asimakis, Bertolini, & Dunstan, 2011; Thomas & Geer, 2011).

The ability to assess aggregation propensity is essential for any biopharmaceutical, including mAb‐based products, to progress successfully from molecule to market (Jain et al., 2017; Tiller & Tessier, 2015; van der Kant et al., 2017). In silico analyses, such as TANGO (Fernandez‐Escamilla, Rousseau, Schymkowitz, & Serrano, 2004), Zyggregator (Tartaglia & Vendruscolo, 2008), Waltz (Oliveberg, 2010), Aggrescan (Conchillo‐Solé et al., 2007), and PASTA (Trovato, Seno, & Tosatto, 2007) can be used to predict the presence of aggregation‐prone regions (APRs) within proteins using protein primary sequence information alone. In addition, CamSol (Sormanni, Aprile, & Vendruscolo, 2015) and SAP can be used to predict aggregation‐prone (poorly soluble) surface‐exposed regions (Chennamsetty, Voynov, Kayser, Helk, & Trout, 2009; Trainor, Broom, & Meiering, 2017) while Solubis (Van Durme et al., 2016) integrates thermodynamic data, allowing identification of buried APRs that may be transiently exposed (van der Kant et al., 2017). A caveat of these latter in silico methods is the necessity of high resolution structural data for the molecule under study. These developing in silico approaches complement established accelerated stress studies that are performed in vitro to predict the shelf‐life and stability of biologics (Jain et al., 2017; Yang et al., 2013). Various methods are employed to generate such data including heating (Cheng et al., 2012; Hamrang et al., 2015), stirring (Luo et al., 2011; Sediq, Van Duijvenvoorde, Jiskoot, & Nejadnik, 2016), shaking (Kiese, Papppenberger, Friess, & Mahler, 2008; Rudiuk, Cohen‐Tannoudji, Huille, & Tribet, 2012), and simulation of transportation (Fleischman, Chung, Paul, & Lewus, 2017). The extent of aggregation, however, can be heavily dependent on the type of accelerated stress employed (Fleischman et al., 2017; Joubert, Luo, Nashed‐Samuel, Wypych, & Narhi, 2011; Tamizi & Jouyban, 2016).

**Figure 1:**
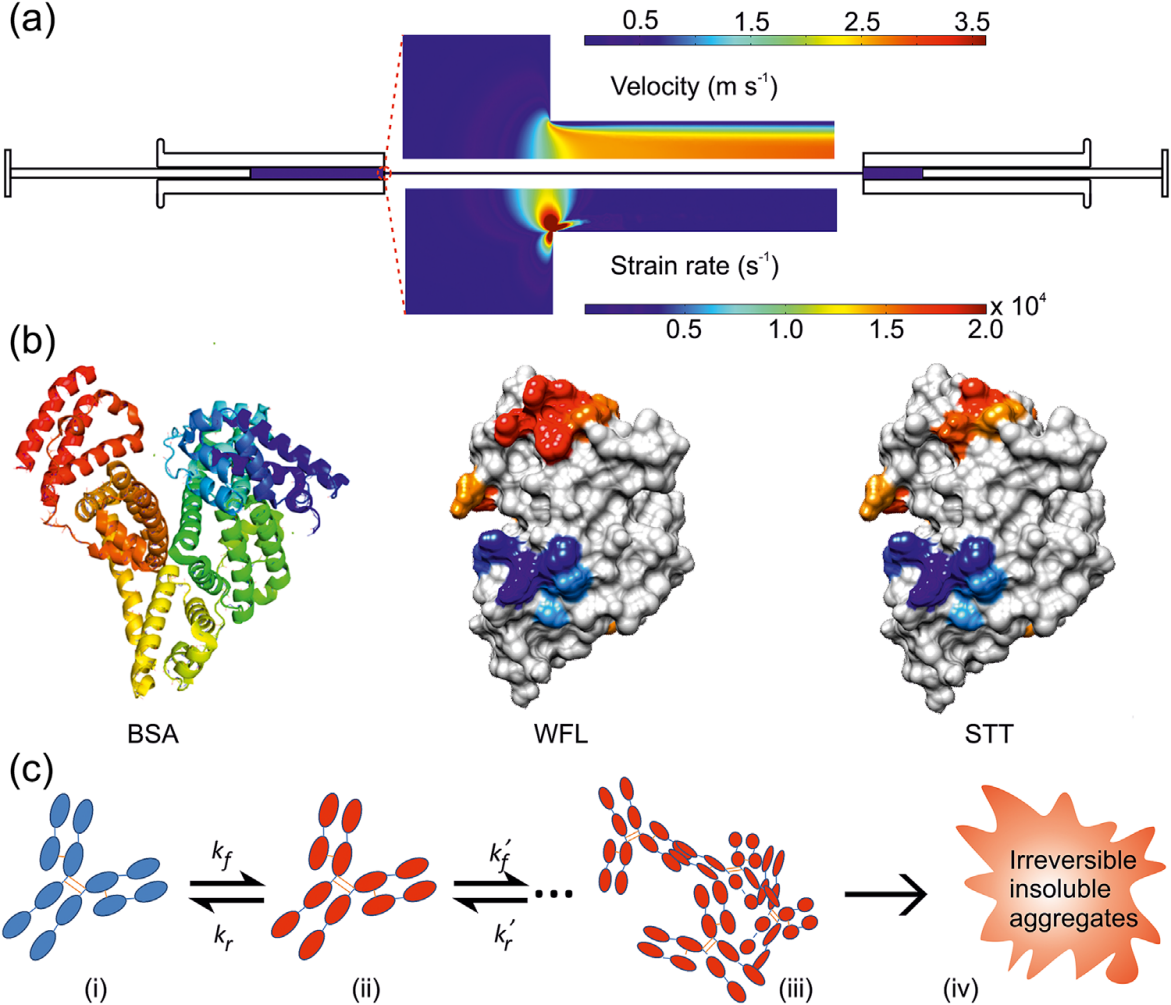
Extensional flow‐induced protein aggregation. (a) Schematic of the extensional flow device (EFD). Protein solution is driven from the syringe (left) though the capillary via a stepper motor. The fluid velocity is slow and laminar in the syringe. The abrupt contraction in the flow, where the capillary connects the syringes, causes a rapid increase in fluid velocity, generating an extensional flow. A high shear flow is then established along the length of the capillary. Computational fluid dynamics profiles (Dobson et al., 2017) of velocity (top) and strain rate (bottom) highlight the extensional flow field. The protein solution then enters the other syringe, ready to be shuttled back on the next pass. (b) Structures of BSA (PDB ID: 3V03) (Majorek et al., 2012), WFL and STT. The surface‐view scFv models of WFL and STT were built from the structure of their parent, MEDI_578 (PDB ID: 5jz7) (Dobson et al., 2016). The surface for each protein is color‐coded according to the CamSol structurally corrected solubility profiles of WFL and STT to show regions of poor solubility (red) and enhanced solubility (blue) (Supplementary Methods). (c) Proposed mechanism of flow‐induced mAb aggregation. (i) The native protein (blue) is perturbed into an aggregation‐prone state (red), the relative level of which is dependent on the fluid field and the protein studied. (ii) This can either re‐fold to the native state or proceed along the aggregation pathway (iii) to form irreversible, insoluble aggregates (iv). The apparent rate constants (*k_f_* and *k_r_*) represent the rate of formation/refolding of the aggregation‐prone state, while *k_f_^’^* and *k_r_^’^* represent the concentration‐dependent rate of oligomer formation and the unimolecular off‐rate for oligomer dissociation, respectively. These rate constants remains to be determined

Differences in accelerated and innate aggregation propensities (Goldberg et al., 2017) may arise because the acceleration method increases the relative flux through certain pathways which are distinct to those traversed during production or upon storage (Chakroun, Hilton, Ahmad, Platt, & Dalby, 2016; Luo et al., 2011; Phillips et al., 2017; van der Kant et al., 2017). There is thus a need to develop stress tests that more closely replicate the conformational ensemble generated during processing and transport. In light of this, we and others have shown that hydrodynamic extensional flow fields encountered during the nano‐filtration, pumping, and fill‐finish steps of bio‐processing can trigger protein aggregation (Charm & Wong, 1981; Dobson et al., 2017; Simon, Krause, Weber, & Peukert, 2011; Wolfrum, Weichsel, Siedler, Weber, & Peukert, 2017). Using a reciprocating extensional and shear flow device (EFD) (Figure 1a), we showed that extensional flow fields can induce the conformational unfolding/remodeling of bovine serum albumin (BSA, Figure 1b), leading to aggregation that was characterized and quantified by an array of biophysical techniques including DLS, NTA, and TEM (Dobson et al., 2017). By subjecting five other globular proteins that varied in sequence, size, and structure to such flow stresses, we demonstrated that the aggregation propensity of proteins differed based on their fold, sequence, and the fluid fields to which they are subjected (Dobson et al., 2017). For mAb‐based biotherapeutic scaffolds we observed a wide‐range of sensitivity to flow‐induced aggregation under identical conditions (strain rate, pass number, protein concentration and buffer), dependent on the protein sequence (Dobson et al., 2017). Accordingly, the aggregation‐prone antibody (MEDI1912_WFL, WFL herein, Figure 1b) was found to be most sensitive to extensional flow, while its rationally engineered aggregation‐resistant derivative (MEDI1912_STT, STT herein, Figure 1b (Dobson et al., 2016) showed markedly decreased aggregation (∼85 and ∼5% aggregation, respectively). An unrelated mAb, mAb1, showed intermediate behavior (Dobson et al., 2017). For BSA, the extent of aggregation is dependent on the magnitude of the strain rate, the total exposure time to the extensional flow event (controlled by both the number of passes and plunger velocity) and the protein concentration, producing a complex aggregation landscape. These observations suggest that the flow‐induced aggregation of mAbs may proceed via a common mechanism, resulting in the formation of insoluble, amorphous aggregates (Figure 1c). Here, using the EFD and a protein pelleting assay, we have mapped in detail the aggregation landscape of WFL, STT, and mAb1 after exposure to defined fluid fields, exposure times, and protein concentrations. These experiments reveal distinct behavior under flow for these structurally similar proteins. Additionally, we show that the same protein can exhibit diverse behavior under flow, dependent on the buffer used. Quantifying extensional flow‐induced aggregation of mAbs may thus allow the rational selection of more robust biopharmaceuticals and the identification of buffer and manufacturing conditions which minimize aggregation.

## 2 MATERIALS AND METHODS

### 2.1 Protein sample preparation

The proteins used in the study (WFL, STT, and mAb1) were provided by MedImmune Ltd, Cambridge UK, as described previously (Dobson et al., 2016, 2017). Buffer reagents were obtained from Sigma–Aldrich (Gillingham, UK), except sodium phosphate dibasic (BDH Lab Supplies, Bristol, UK) and L‐arginine (Acros Organics, Geel, Belgium). In all experiments, antibodies were dialyzed into the appropriate 0.22 μm‐filtered and de‐gassed buffer and subsequently used in extensional flow experiments. Bovine Serum Albumin (BSA) was prepared as described previously (Dobson et al., 2017). Except for the buffer screen, all mAb experiments were performed in 150 mM ammonium acetate buffer, pH 6.0. For experiments involving BSA, 25 mM ammonium acetate buffer pH 5.1 was used.

### 2.2 Extensional flow device (EFD) and stress experiments

Full details of the extensional flow device including its validation using computational fluid dynamics (CFD) are described elsewhere (Dobson et al., 2017). Briefly, the EFD consists of two modified Hamilton gas‐tight syringes (inner diameter = 4.6 mm) connected by a 0.3 mm inner‐diameter borosilicate glass capillary. The capillary length was 75 mm in all experiments except shear‐length variation experiments, where a ceramic cutter was used to shorten capillaries to 50 mm (2/3 length) or 37.5 mm (1/2 length) followed by flame‐finishing. All protein solutions were 0.22 μm‐filtered prior to loading into the device, and any air‐bubbles ejected prior to assembling the EFD. The protein solution was shuttled between the syringes at the desired plunger velocity (determining the strain rate and the shear rate) for a given number of passes (determining exposure time); see Supplementary Table S1 for plunger velocities and concomitant center‐line strain and shear rates. The plungers were driven by a stepper motor controlled by an Arduino microcontroller. After subjecting the protein to the desired number of passes, the EFD was dissembled and the protein solution removed for quantification of aggregation (insoluble protein pelleting assay, below). All experiments were performed at a concentration of 0.5 mg ml^−1^ for mAbs and 5 mg ml^−1^ for BSA unless stated otherwise. As a control, a sample was incubated under ambient conditions (quiescent) alongside the stressed sample for the duration of the experiment and subsequently subjected to the same analysis.

The buffer screen was carried out in five buffers: 10 mM L‐histidine pH 6.0; 10 mM sodium acetate pH 6.0; 10 mM sodium succinate pH 6.0; 10 mM sodium phosphate pH 7.2; and 125 mM L‐arginine + 20 mM sodium succinate pH 6.0.

### 2.3 Insoluble protein pelleting assay

The insoluble protein formed after stress in the flow device was quantified using an insoluble protein assay (Dobson et al., 2017). Briefly, 2 × 200 μl of the protein sample under test (stressed or quiescent) was centrifuged at 30,000 rpm in a Beckmann Coulter Optima TLX ultracentrifuge, equipped with a TLA100 rotor for 30 min. A total of 150 μl of supernatant was then removed from each tube. A total of 200 μl 6 M guanidine hydrochloride buffer, pH 6.0 was added to 50 μl of this supernatant (giving [*S*] after quantification, Equation (1)) and to the ∼50 μl solution (including the insoluble fraction) that remained in the ultracentrifuge tube (giving [*P*] after quantification, Equation (1)) and incubated at 4°C overnight. The concentration of solubilized protein was quantified using UV spectroscopy at 280 nm using extinction coefficients of 43824, 207360, 239440, and 228440 M^−1^ cm^−1^ for BSA, mAb1, WFL, and STT, respectively. The % pelleted protein was calculated using equation (1):

(1) $$\%\;\text{ protein in pellet=}(\frac{\left(\left[P]-[S\right]\right)}{\left({\left[\text{protein}\right]}_{0}\right)})\times 100$$

where [*P*] is the concentration of protein in the pellet fraction, [*S*] is the concentration of protein in the supernatant fraction and [protein]_0_ is the initial protein concentration.

Data were analyzed and plotted using Microsoft Excel 2015 and Origin 2017, respectively.

### 2.4 Supplementary online material

Supplementary Methods describe 3D aggregation landscape plotting, intrinsic solubility calculations using CamSol and a table that lists the center‐line strain rate and capillary wall shear rate associated with each plunger velocity. Supplementary Results show the effect of low strain rates in the aggregation of WFL and STT.

## 3 RESULTS

### 3.1 Mapping the aggregation behavior of WFL and STT

**Figure 2:**
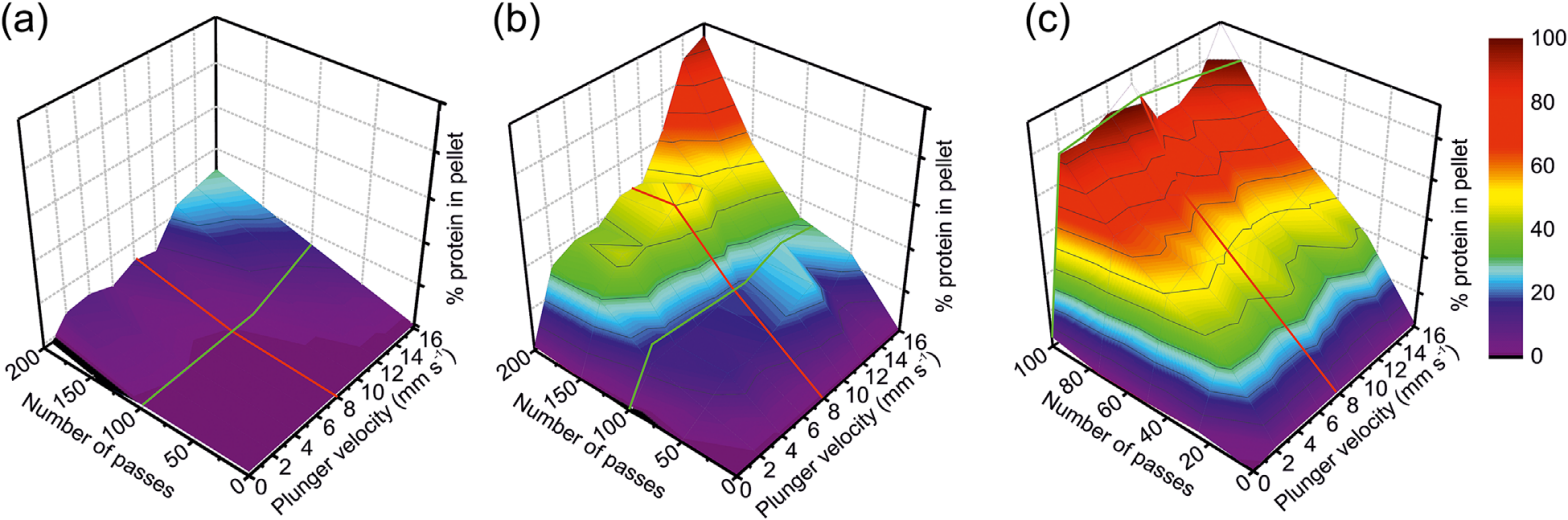
Aggregation landscapes of (a) BSA, (b) STT, and (c) WFL. The 3D surface plots show the percentage of insoluble protein formed after stress in the flow device for the given number of passes at a range of strain rates (3,184–23,421 s^−1^; see Supplementary Table S1). (a) BSA was stressed for 200 passes at a concentration of 5 mg ml^−1^ in 25 mM ammonium acetate, pH 5.1. These data were plotted alongside the data obtained for 100 passes from (Dobson et al., 2017). Total number of data points = 18 (including quiescent samples). (b and c) The two antibodies (STT and WFL, respectively) were stressed at a concentration of 0.5 mg ml^−1^ in 150 mM ammonium acetate buffer, pH 6.0. Total number of data points = 45 per surface (including border from quiescent samples). Red lines indicate the response to increasing passes at a plunger velocity of 8 mm s^−1^. Green lines indicate the response to increasing strain rate and 100 passes through the device. All data shown are the average of two independent experiments

We showed previously that the extent of flow‐induced aggregation of different proteins is sensitive to the strain rate and exposure time (and protein concentration) when modulated independently. For example, for BSA, a center‐line strain rate of at least 14,634 s^−1^ (a plunger speed of 10 mm s^−1^) was required before significant quantities of aggregated protein could be detected after 100 passes (data along green line, Figure 2a and Dobson et al., 2017). This suggests that a force threshold, applied from the flow onto the protein in the extensional flow field, has to be overcome to induce local unfolding and subsequent aggregation of the protein. However, as partial unfolding is assumingly a stochastic process, the extent of aggregation was also found to be pass number dependent (red line, Figure 2a). To investigate the co‐dependence of these variables, BSA, WFL, and STT were stressed for different numbers of passes at different plunger velocities (Methods and Supplementary Methods). The amount of insoluble protein formed under each flow condition was quantified and the data then plotted as a three‐dimensional response surface (Figure 2a–c and Supplementary Methods). As observed previously, it is clear from the three surfaces that each protein displays distinct sensitivity to flow‐induced aggregation, with BSA most resistant and WFL most sensitive. BSA, the aggregation of which (at a plunger velocity of 8 mm s^−1^) was characterized in detail in our previous study (Dobson et al., 2017), has a relatively flat aggregation landscape (Figure 2a); below 10 mm s^−1^, little aggregation is induced, irrespective of pass number. Above this plunger velocity, aggregation becomes dependent on both the plunger velocity and pass number, so that after 200 passes at 16 mm s^−1^, ∼30% of BSA is rendered pelletable STT displays a similar profile (Figure 2b) but with a greater aggregation propensity. After 50 passes, STT shows little aggregation over the investigated range of plunger velocities (strain rates), and the increase in aggregation with the number of passes is small at slow plunger velocities. The aggregation landscape of STT is relatively flat and low in magnitude in regions where the strain rate and/or pass number is small. As the number of passes and plunger velocity increases, the rise in aggregation is significantly greater than for BSA (e.g., 95% of STT is insoluble after 200 passes at a plunger velocity of 16 mm s^−1^). This concurs with the hypothesis that traversing the extensional flow region of the device promotes the population of transient aggregation‐prone, partially unfolded species that then interact, triggering aggregation. The relative population of these species over the time course of the experiment may be increased by greater energy input (faster flows) or by increasing the number of opportunities for partial unfolding to occur (increased pass number).

In contrast to these data, the response surface of WFL shows only a simple monotonic profile (Figure 2c), where aggregation is dependent solely on pass number above 2 mm s^−1^. For example, at 8 mm s^−1^ aggregation increases from ∼35 to 100% upon increasing from 20 to 100 passes (red line, Figure 2c). Despite the velocity independence, hydrodynamic flow is still necessary to induce aggregation, as minimal aggregation is observed under quiescent conditions. Above the 2 mm s^−1^ threshold, the number of passes through the device dictates the amount of aggregate observed (∼90–100% after 100 passes). For WFL, these data suggest that (i) the aggregation‐prone patches of the protein are exposed to the solvent upon experiencing a relatively small strain rate (6,031 s^−1^) or that (ii) the protein maintains its aggregation‐prone state after traversing the extensional flow region or that (iii) the affinity/on‐rate for WFL oligomerization is higher relative to STT.

For both WFL and STT, not all strain rates bring about aggregation. When WFL was stressed at 0.5 mm s^−1^ for 20 passes, minimal aggregation was observed, whereas when STT was stressed for 50 passes at 0.5 mm s^−1^ no aggregation was observed (Figure S1). Flow‐induced protein aggregation thus appears to be a complex function of the force that the protein is exposed to and the number of times the protein experiences exposure to this force, convoluted with the on‐ and off‐rates of aggregation. This multi‐factorial dependence results in distinct aggregation landscapes for even very closely related proteins.

### 3.2 STT and WFL exhibit different concentration dependencies of aggregation

**Figure 3:**
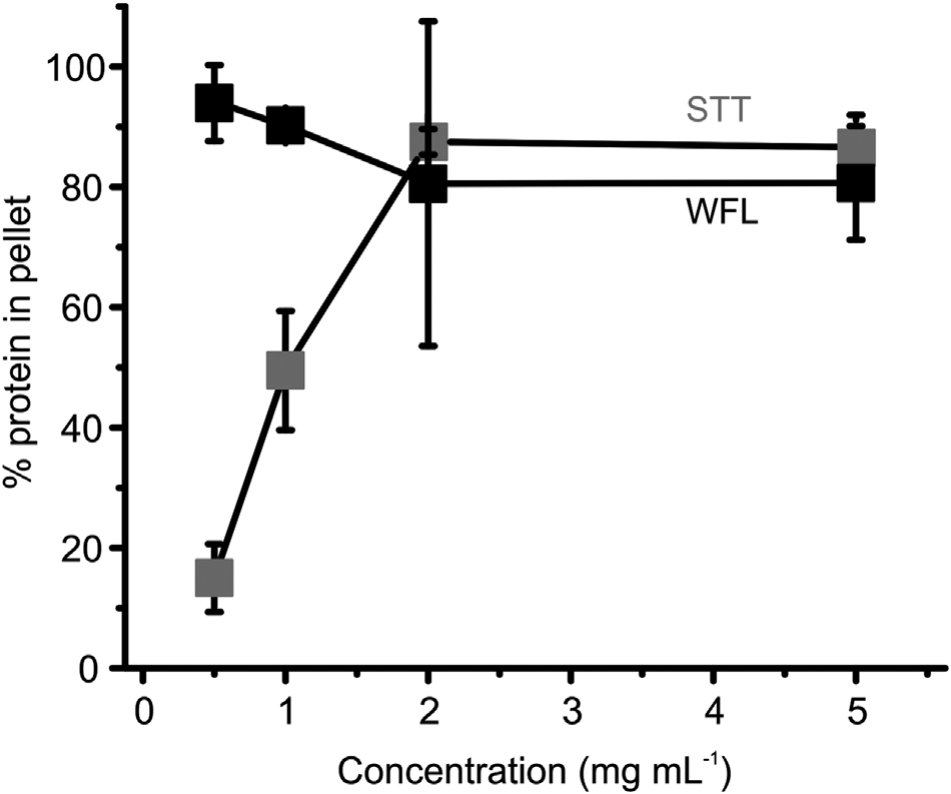
Effect of protein concentration on WFL and STT aggregation under flow stress. WFL and STT were stressed for 100 passes at a plunger velocity of 8 mm s^−1^ over a range of concentrations (0.5, 1, 2, and 5 mg ml^−1^) in 150 mM ammonium acetate pH 6.0, then analyzed with the pelleting assay. Points represent the relative amount of insoluble WFL (black squares) and STT (gray squares). Error bars indicate the error from two independent experiments

The data above suggest a mechanism whereby aggregation is driven by inter‐molecular collisions between activated species and/or with ground‐state proteins. Next, we sought to examine whether increased protein concentration resulted in increased aggregate formation under equivalent flow conditions, as expected from such a model. Accordingly, WFL and STT were stressed for 100 passes at a concentration of 0.5, 1, 2, and 5 mg ml^−1^ at a plunger velocity of 8 mm s^−1^ and the extent of aggregate formation was quantified. Once more, STT and WFL showed diverse behavior (Figure 3). For STT, protein concentration strongly affected the aggregation probability, increasing from 15 to 88% at 0.5 and 2 mg ml^−1^, respectively. After this point, no further increase in the percent of aggregation was observed, suggesting that at a concentration of 2 mg ml^−1^ or higher, the aggregation reaction had reached its end point by 100 passes. WFL, by contrast, showed almost complete aggregation (94%) at a concentration as low as 0.5 mg ml^−1^. WFL and STT only differ by three residues in each V_H_ domain (two in CDR1 and one in CDR2) (Dobson et al., 2016). The intrinsic solubility scores (i.e., based on primary sequence alone) of CDRs 1 and 2, calculated using CamSol, yields values of 0.005 and 0.302 for WFL and 1.347 and 0.505 for STT, respectively (Supplementary Methods). The CDR1 of STT is therefore predicted to be markedly more soluble (greater positive CamSol score) than the same region in WFL (Sormanni et al., 2015). These data suggest either that flow perturbs the structure of these CDRs, exposing aggregation‐prone regions to the solvent to different extents for WFL and STT, and/or that the exposed APRs have different affinities. Previous cross‐linking studies under quiescent conditions have shown that WFL homodimer formation occurs via V_H_‐V_H_ interactions, with the binding interface comprising residues 30, 31 (CDR1), and 56 (CDR 2) (Dobson et al., 2016). Whether the same interface is formed under extensional flow remains to be seen.

### 3.3 Aggregation‐prone mAbs are sensitive to high shear

**Figure 4:**
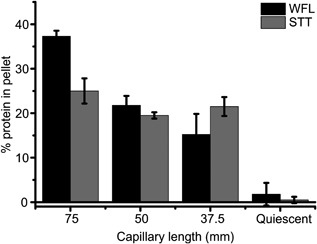
Shear‐length dependence on WFL and STT aggregation. Decreasing the length of the borosilicate glass capillary decreases the length of time proteins are exposed to high shear downstream of the extension region. WFL was stressed for 20 passes at a plunger velocity of 8 mm s^−1^, while STT was stressed for 100 passes at the same plunger velocity. The capillary wall shear rate in the capillary = 50,375 s^−1^. Bars show percentage insoluble WFL (black bars) and STT (gray bars) formed following stress using capillaries of differing length. Capillary length was either 75 mm (full‐length), 50 mm (2/3), or 37.5 mm (half‐length). The quiescent samples were not exposed to flow stress. Both proteins were stressed at a concentration of 0.5 mg ml^−1^ in 150 mM ammonium acetate buffer, pH 6.0. Error bars indicate the error from two independent experiments

The EFD generates a well‐defined region of extensional flow at the point of contraction, followed by shear flow along the length of capillary (Figure 1a) (Dobson et al., 2017). Shear flow within the capillary (characterized by the shear rate) could thus play a role in aggregation, despite a much smaller rate of energy transfer per unit time (28 (E/*k_B_*T.t) s^−1^ vs 150,000 (E/*k_B_*T.t) s^−1^ for the shear and extension regions respectively at a plunger velocity of 8 mm s^−1^ (Dobson et al., 2017)). For BSA, this possibility was obviated as aggregation was found to be independent of capillary length, using flow conditions under which BSA is most susceptible to aggregation (plunger velocity of 16 mm s^−1^) (Dobson et al., 2017). The data for WFL and STT above, however, show that the aggregation landscapes for mAbs differ from that of stable, globular proteins such as BSA. To determine the effect of shear flow on mAb aggregation, WFL and STT were subjected to 20 and 100 passes (respectively, to generate a similar aggregate yield) at a plunger velocity of 8 mm s^−1^, using capillary lengths of 37.5, 50, and 75 mm (Methods). The extent of aggregation was then quantified using the pelleting assay. Figure 4 shows that the aggregation of STT is independent of capillary length, but that WFL aggregation decreases from 37% to 15% upon halving the capillary length, indicating that shear flow contributes to the aggregation of this protein. Further work is required to identify the underlying mechanism behind this observation. For example, this effect may arise because the key aggregation‐prone region of WFL may be exposed at a lower strain rate threshold or because the presence of a shear region may slow the relaxation time of the activated species. Irrespective of the mechanism, the results show that these highly homologous mAbs display diverse behavior under flow.

### 3.4 mAb1 exhibits traits of both WFL and STT in response to flow

**Figure 5:**
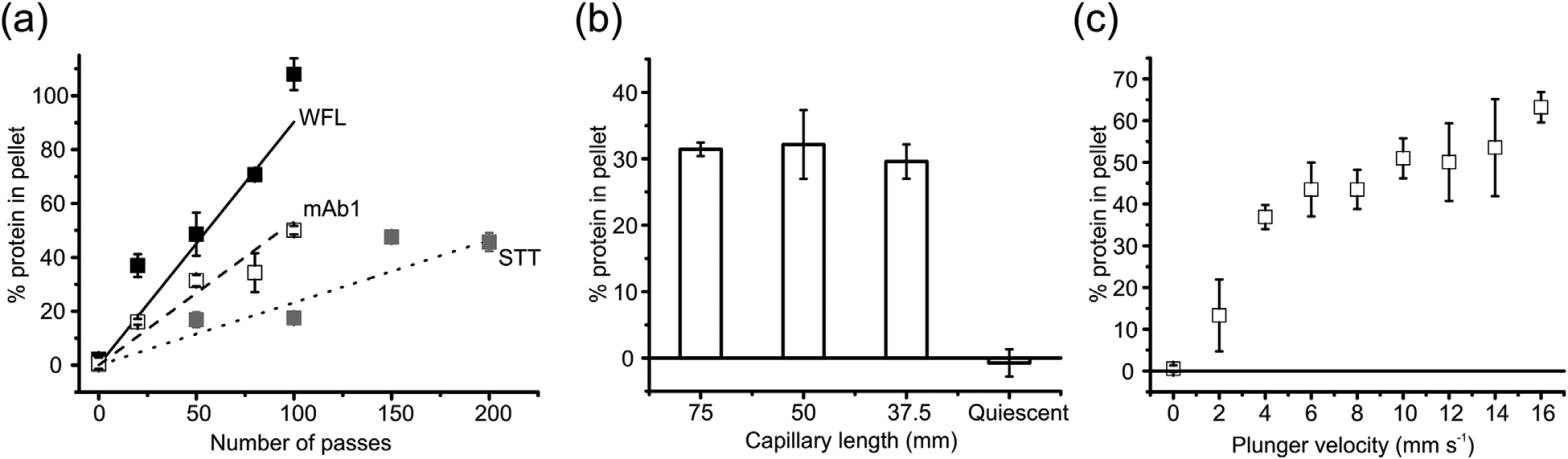
Aggregation behavior of mAb1 under different flow conditions. (a) Plot of percentage insoluble mAb1 formed as a result of a defined number of passes through the flow device at a plunger velocity of 8 mm s^−1^ (white squares). Data for WFL (▪) and STT (▪) (data taken from red lines in Figures 2c and b, respectively) are shown for comparison. Linear trend lines to guide the eye are shown for WFL (solid black line), mAb1 (dashed line), and STT (dotted line), respectively. (b) Shear length variation on mAb1 stressed for 50 passes at a plunger velocity of 8 mm s^−1^ with capillaries of varying length. Full‐length = 75 mm, 2/3 = 50 mm, and half‐length = 37.5 mm. (c) mAb1 aggregation after 100 passes through the flow device at different plunger velocities (2–16 mm s^−1^). mAb1 was stressed in 150 mM ammonium acetate, pH 6.0 at a concentration of 0.5 mg ml^−1^ in all experiments. Error bars indicate the error from two independent experiments

While comparing the biophysical properties of STT and WFL under quiescent and flow conditions is an extremely powerful method to delineate the mechanism and determinants of flow‐induced aggregation, these proteins may represent extremes of the mAb scaffolds usually encountered during bio‐processing. We thus complemented the above data by examining the (plunger velocity (2–16 mms^−1^), pass number (0–100) and shear exposure time (37.5, 50, and 75 mm) dependencies of mAb1, an unrelated IgG1 that has ∼72% sequence identity to WFL and STT (Dobson et al., 2016, 2017). The data in Figure 5a show that, similarly to WFL and STT, the extent of mAb1 aggregation is directly dependent on pass number and that mAb1 displays intermediate sensitivity to pass number (rank order: WFL>mAb1>STT). Like STT (Figure 4), the aggregation of mAb1 is independent of the length of the shear region (31.1 ± 1% and 29.6 ± 2% for full‐ and half‐length capillaries, respectively) (Figure 5b). mAb1 also displays a STT‐like response in aggregation level to an increase in plunger velocity, with low levels of aggregation observed at 2 mm s^−1^ and increased levels of aggregate with increasing strain rate (Figure 5c). Comparison of these three IgG1s reveal that their primary sequence modulates flow‐induced aggregation, producing complex and distinct landscapes dependent on the sequence and flow conditions used.

### 3.5 Using the EFD as a buffer screening tool

**Figure 6:**
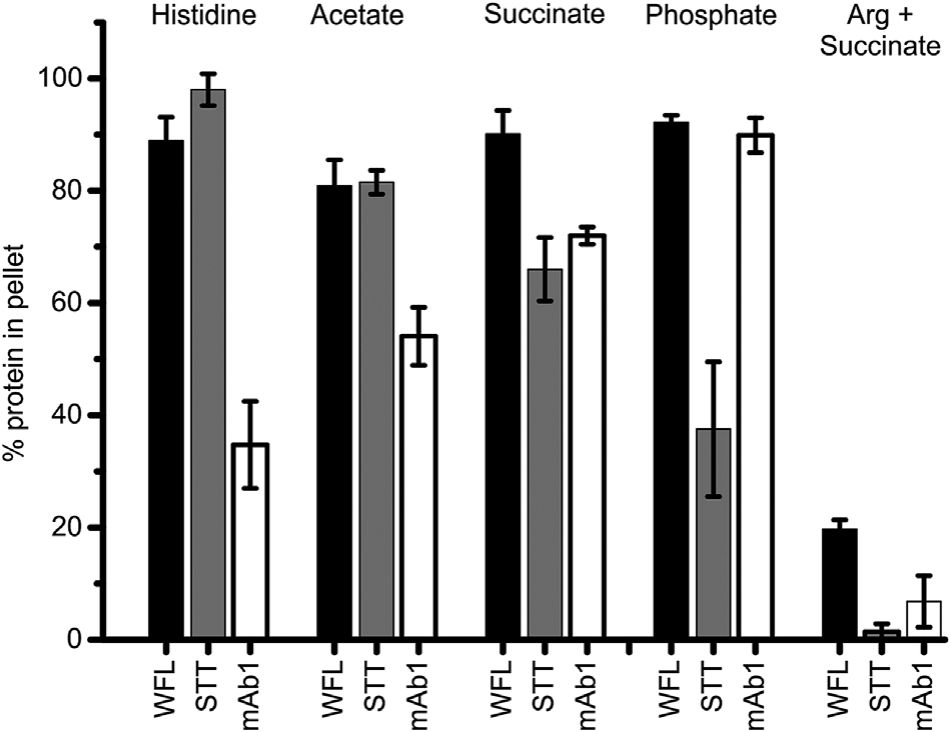
Using the EFD to screen optimal buffers for WFL, STT, and mAb1. WFL (black bars), STT (gray bars), and mAb1 (white bars) were dialyzed into 10 mM L‐histidine pH 6.0, 10 mM sodium acetate pH 6.0, 10 mM sodium succinate pH 6.0, 10 mM sodium phosphate pH 7.2, and 125 mM L‐arginine + 20 mM sodium succinate pH 6.0 and stressed for 100 passes in the device at a plunger velocity of 8 mm s^−1^. Error bars indicate the error from two independent experiments

The buffer composition (ionic strength, buffering salt, and pH) is known to affect the processability and formulated stability of biopharmaceuticals (Dobson et al., 2016; Goldberg et al., 2017; Wang, 2015). To assess the effect of buffer composition on stability under flow, STT, WFL, and mAb1 were dialyzed into four buffers in which the quiescent stability of STT and WFL is known, as well as arginine + succinate buffer (Methods) (Dobson et al., 2016). Each mAb (0.5 mg ml^−1^) was then stressed for 100 passes at 8 mm s^−1^ and the resulting aggregation quantified. The results show that the ability of each buffer to modulate aggregation is dependent on the mAb sequence (Figure 6). For example, the high flow sensitivity of WFL aggregation was largely maintained in all buffers, with arginine + succinate a noteworthy exception (aggregation decreased from ∼86% in histidine, acetate, succinate, and phosphate buffers to 20% in arginine + succinate buffer). Interestingly, both STT and mAb1 displayed buffer‐dependent levels of flow‐induced aggregation. These effects were mAb‐dependent, with STT showing a greater extent of aggregation in histidine compared with phosphate and vice‐versa for mAb1. Given arginine's widespread use as a stabilizing excipient (Baynes, Wang, & Trout, 2005; Kim, Hada, Thapa, & Jeong, 2016), it is also of note that all mAbs exhibit greatly suppressed aggregation in arginine + succinate buffer. Extensional flow‐induced mAb aggregation is thus sensitive to both the buffer used and the mAb sequence. Previous work to identify the buffer which afforded STT the greatest stability used dynamic light scattering. This required higher protein concentrations (4 mg ml^−1^) (Dobson et al., 2016) and showed limited ability to differentiate between buffers. The results presented here suggest that extensional flow stress tests could be used to screen the stability of mAbs in different buffer environments and hence to optimize conditions in the pipeline for biologics production, using as little as 0.25 mg of protein per experiment.

## 4 DISCUSSION

Examining the effect of both the magnitude and duration of defined hydrodynamic stresses on protein solutions has been carried out on several globular proteins (Dobson et al., 2017; Simon et al., 2011). Performing such experiments on proteins of more complex topology and inherent commercial value, such as mAbs, is useful to both the biotechnology industry and the wider scientific community. We show here that the aggregation response of WFL and STT to flow are distinct, despite these mAbs differing by only three residues per V_H_ domain. WFL formed large amounts of insoluble protein under flow, even when exposed to low strain rates for as few as 20 passes. By contrast, the STT aggregation landscape shows a much larger region where few aggregates form. Such an analysis could inform manufacturing practice through relating the strain rates and exposure times experienced in the EFD to plant equipment (e.g., the multiple passes experienced in a tangential flow filtration device (Rosenberg, Hepbildikler, Kuhne, & Winter, 2009)). To do this, studies of plant equipment need to report strain rates due to extensional flow alongside the more commonly reported shear rates (Bee et al., 2009; Charm & Wong, 1981).

It has been suggested that shear flow does not cause damage to the tertiary structure of proteins (Bee et al., 2009), and/or that the shear rates required to do so may be hard to achieve experimentally (Jaspe & Hagen, 2006). We show here, however, that reducing the amount of time the protein is exposed to high shear (by decreasing the length of the capillary), WFL aggregation is diminished, indicating a role for shear in the process (note: at a plunger velocity of 8 mm s^−1^, proteins spend ∼18 µs in the extension region, 40 ms in the capillary, and ∼5 s in the syringes (Dobson et al., 2017)). Whether shear alone or an initial extensional flow event prior to shear is required to induce WFL aggregation remains to be seen. The latter scenario could cause a localized unfolding of a protein molecule (e.g., as seen for von Willebrand factor (Lippok et al., 2016) and for BSA in our previous study (Dobson et al., 2017), which may then be susceptible to “tumbling” events under shear flow in the capillary (Smith, Babcock, & Chu, 1999) and rapid self‐association in a concentration and time‐dependent manner.

The mAb1 antibody exhibited “intermediate” behavior when subjected to the same experiments as WFL and STT. mAb1 showed a linear pass number dependence (similar to WFL and STT) but a strain‐sensitivity and shear‐insensitivity akin to BSA and STT (Figures 2a and 2b). Taken together, our experiments point toward a common mechanism of mAb aggregation induced by the flow fields present in our device (Figure 1c) centered on the formation of activated, aggregation‐prone species that readily self‐associate, forming soluble, and then insoluble aggregates (Dobson et al., 2017; Roberts, 2007; Wang, Nema, & Teagarden, 2010). The flux through the pathway is governed by the ability of extensional flow to activate each native mAb into a perturbed structural state (van der Kant et al., 2017), the affinity of the exposed APR, the rate of relaxation from the activated species (which may itself be modulated by force [Bustamante, Chemla, Forde, & Izhaky, 2004]) and the productive collisional frequency. Consequently, mechanically robust proteins such as BSA (seventeen intra‐molecular disulfide cross‐links in the native state) or, like STT, those with reduced aggregation propensity, require the application of high strain rates and/or pass number to induce appreciable aggregation.

The aggregation behavior of mAbs under hydrodynamic stress is clearly affected by a variety of parameters. The defined nature of the flow environment in the EFD can allow the effects of protein sequence and concentration, surface chemistry (Biddlecombe et al., 2007), strain rate, shear rate and the total exposure time on the observed aggregation to be determined in the absence of other confounding factors such as air‐water interfaces or the action of stirring (Fleischman et al., 2017; Joubert et al., 2011; Kiese et al., 2008; Tamizi & Jouyban, 2016; Zhao & Cieplak, 2017). Furthermore, by subjecting mAbs to hydrodynamic stress in different buffer conditions, we have highlighted that the choice of excipient can be another crucial factor in influencing the extent of aggregation, opening up the possibility of using the EFD as a formulation tool.

In summary, by mapping the response of three mAbs to defined hydrodynamic flows, we have demonstrated that the aggregation of these proteins can be minimized by changing processes (e.g., operating under low strain conditions), changing sequence (WFL vs. STT), or by changing buffer conditions.

## 5 CONCLUSION

There is an unmet need to predict the aggregation propensity of proteins of pharmaceutical interest, at an early stage in the development pipeline, in order to maximize their chances of success of transitioning from the bench to the market (Jain et al., 2017). Various accelerated stress methodologies, such as stirring, heating, and shaking, are employed to make such assessments (Tamizi & Jouyban, 2016). Nonetheless, ranking the probability of failure during bioprocessing remains a significant challenge. Here, we use a well‐characterized extensional and shear flow device to subject three mAbs to defined fluid fields (strain rate, shear rate, and exposure time). Quantifying the resultant aggregation, allows estimation of the likelihood of biopharmaceutical aggregation under flow and the identification of buffers that minimize the effects of flow. This method will be a useful addition to the repertoire of tools available to the biopharmaceutical industry to distinguish “manufacturable” proteins from poorer candidates, requiring only small quantities of protein and thus allowing assessment early in the development pipeline.

## CONFLICTS OF INTEREST

The authors declare no conflicts of interest.

## Supporting information

Additional Supporting Information may be found online in the supporting information tab for this article.

Supporting Data S1.
