# Supplementary material for: Using extensional flow to reveal diverse aggregation landscapes for three IgG1 molecules
Source: Biotechnol Bioeng. 2018 Feb 4;115(5):1216–25. doi: 10.1002/bit.26543 (PMC5900942; doi:10.1002/bit.26543)
Supplement: Supplementary file 1 — Supporting Data S1. [file BIT-115-1216-s001.docx]

**Supplementary Information**

Using extensional flow to reveal diverse aggregation landscapes for three IgG1 molecules

Leon F. Willis, Amit Kumar, John Dobson, Nicholas J. Bond, David Lowe, Richard Turner , Sheena E. Radford, Nikil Kapur and David J. Brockwell

**Supplementary Methods**

**3D aggregation landscape plots.** BSA was stressed for 200 passes over a range of plunger velocities (2–16 mm s^-1^), then the amount of insoluble protein quantified by the pelleting assay (see main Methods and Figure 2A). These data were plotted alongside those obtained after 100 passes in our previous study (Dobson et al. 2017) (Figure 2A). STT was stressed for 50, 100, 150 and 200 passes over range of plunger velocities stated above, before the amount of insoluble protein was quantified using the pelleting assay (Figure 2B). WFL was stressed for 20, 50, 80 and 100 passes at the plunger velocities above, before being subjected to the same analysis (Figure 2C). The quiescent BSA samples showed negative values by pelleting (black shading along passes axis), meaning no aggregation occurred. The border values along the strain axis values were thus set to zero. The quiescent WFL and STT sample values were averaged and represent the borders on each surface (purple). The data were converted into 3D matrix form and plotted as a 3D Color map surface in Origin 2017.

**CamSol of WFL and STT.** The CamSol intrinsic solubilities were calculated for the CDR 1 and CDR 2 sequences of WFL and STT, using the CamSol Webserver (Version 2.1) (Sormanni et al., 2015). To generate the structurally corrected solubility structures of WFL and STT at pH 6.0, scFv models from the parent IgG (MEDI_578 (PDB ID: 5jz7) (Dobson et al., 2016)) were created in PyMol and the .pdb files input into the CamSol Webserver. The output structures were generated using the UCSF Chimera visualization package (University of California, San Francisco (supported by NIGMS P41-GM103311)) (Figure 1B).

**Supplementary Table I:** Plunger velocities in the device and the corresponding center-line strain rates.

| **Plunger Velocity (mm s^-1^)** | **Center-line strain rate (s^-1^)** | **Capillary wall shear rate (s^-1^)** |
| --- | --- | --- |
| **0.5** | **871** | **3149** |
| **2** | **3184** | **12594** |
| **4** | **6031** | **25188** |
| **6** | **8887** | **37782** |
| **8** | **11750** | **50375** |
| **10** | **14634** | **62969** |
| **12** | **17555** | **75563** |
| **14** | **20481** | **88157** |
| **16** | **23421** | **100751** |

**
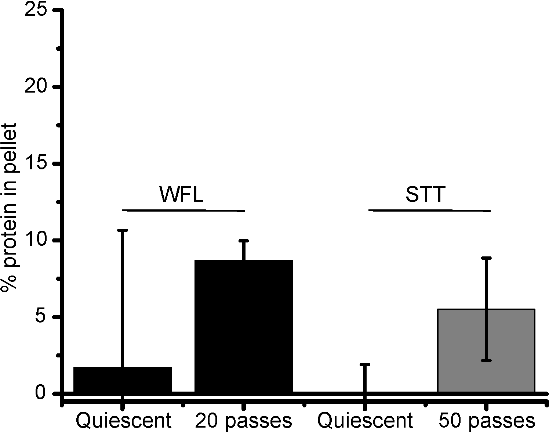
**

**Figure S1. Effect of low strain rates in the aggregation of WFL and STT.** Insoluble protein formed after 20 or 50 passes through the EFD at a plunger velocity of 0.5 mm s^-1^ for WFL and STT, respectively (center-line strain rate = 871 s^-1^). Both mAbs were stressed at a concentration of 0.5 mg mL^-1^ in 150 mM ammonium acetate buffer, pH 6.0.

**References:**

Dobson CL, Devine PWA, Phillips JJ, Higazi DR, Lloyd C, Popovic B, Arnold J, Buchanan A, Lewis A, Goodman J, van der Walle CF, Thornton P, Vinall L, Lowne D, Aagaard A, Olsson L-L, Ridderstad Wollberg A, Welsh F, Karamanos TK, Pashley CL, Iadanza MG, Ranson NA, Ashcroft AE, Kippen AD, Vaughan TJ, Radford SE, Lowe DC. 2016. Engineering the surface properties of a human monoclonal antibody prevents self-association and rapid clearance in vivo. *Sci. Rep.* **6**:38644.

Sormanni P, Aprile FA, Vendruscolo M. 2015. The CamSol method of rational design of protein mutants with enhanced solubility. *J. Mol. Biol.* **427**:478–490.
